# Alterations to unionid transformation during agricultural and urban contaminants of emerging concern exposures

**DOI:** 10.1007/s10646-023-02645-8

**Published:** 2023-04-20

**Authors:** Lacey D. Rzodkiewicz, Mandy L. Annis, Daelyn A. Woolnough

**Affiliations:** 1 Department of Biology and Institute for Great Lakes Research, Central Michigan University, 1455 Calumet Ct., Mt. Pleasant, MI 48859 USA; 2 US Fish & Wildlife Service, Michigan Ecological Services Field Office, 2651 Coolidge Road, Suite 101, East Lansing, MI 48823 USA; 3 Present Address: Department of Biological Sciences, University of Pittsburgh, 4249 Fifth Ave, Pittsburgh, PA 16509 USA

**Keywords:** Life-stage modelling, Freshwater mussels, Obligate parasite, Conservation

## Abstract

Highly imperiled unionids have a complex life cycle including the metamorphosis of an obligate parasite life stage, larval glochidia, to the juvenile stage. Despite the known vulnerabilities of both glochidia and juveniles to pollutants, little is known on how metamorphosis success may be affected by chemical stress. Disruption of the transformation process in which glochidia encyst on the gills of a host fish, could lead to lowered recruitment and population declines. Transformation rates of *Lampsilis cardium* on host fish *Micropterus salmoides* were empirically derived from experimental exposures to low, medium, or high concentrations of an agricultural or urban mixture of contaminants of emerging concern (CECs) over two exposure durations. Transformation was characterized by: (1) a zero-inflated Poisson general linear mixed effects model to compare difference in transformation between exposure durations and (2) time response curves to describe the transformation curve using long-term exposure data. *Lampsilis cardium* transformation was similar between exposure durations. When compared to controls, CEC stress significantly reduced juvenile production (*p* « 0.05) except for the agricultural medium treatment and tended to increased encapsulation duration which while statistically insignificant (*p* = 0.16) may have ecological relevancy. Combining the empirically derived reduction of transformation rates with parameters values from the literature, a Lefkovich stage-based population model predicted strong declines in population size of *L. cardium* for all treatments if these results hold in nature. Management focus on urban CECs may lead to best conservation efforts though agricultural CECs may also have a concentration dependent impact on transformation and therefore overall recruitment and conservation success.

## Introduction

Native freshwater mussels (family: Unionidae), also known as unionids, are a highly imperiled taxon. Approximately 30 species of unionid in North America have been driven to extinction in the last century; globally, 45% of unionid species are near-threatened, threatened, or extinct (Haag and Williams, 2014, Lopes-Lima et al., 2018). Anthropogenic influence on habitat quality and availability are often cited as key contributors to unionid decline, and the influence of pollutants has been identified as a necessary area of focus for unionid conservation (Haag and Williams, 2014, Ferreira-Rodríguez et al., 2019). Unionids are highly sensitive to pollutants such as heavy metals and legacy pesticides (Salazar and Salazar, 1996, Jacobson et al., 1997). Additionally, unionids may experience heightened chemical impacts due to their sessile nature and interaction with both water and soil chemistry, encompassing multiple exposure routes (Newton and Cope, 2007). Unionids used in toxicological studies, such as ours, often share high phylogenetic relatedness with and occupy the same habitats as globally rare unionid species (e.g., Burlakova et al., 2011, Galbraith et al., 2015, Woolnough et al., 2020). Assuming conservation of responses within clades, common unionids can then be used to assess potential stressor impacts and inform conservation of the most imperiled unionid species.

Contaminants of emerging concern (CECs) are a broad group of recently detected pollutants, including current use pesticides, pharmaceuticals, personal care products, and industrial byproducts. In the United States, the US Environmental Protection Agency has not yet set forth regulations on allowable levels or established effects concentrations on aquatic organisms for many CECs, despite their ubiquity (Ankley et al., 2008, Woolnough et al., 2020). Across the Laurentian Great Lakes basin, 93% of 709 water samples assessed contained at least one CEC (Baldwin et al., 2016) and CECs are found in variable combinations in water, sediment, and animal tissue (Woolnough et al., 2020). Sources of these contaminants are numerous and can be attributed to both agricultural and developed land use though the identity of CECs varies by environment (Elliott et al., 2018, Kiesling et al., 2019). Furthermore, CECs are present in intricate mixtures (Elliott et al., 2018). Single contaminant studies of CECs neglect the myriad of possible interactions and thus may not accurately reflect the exposure profiles organisms experience in nature.

Current research, however, on unionid response to CECs and other similar contaminants has focused mainly upon single stressor exposures with emphasis on single life stages (e.g., glochidia or juveniles) (e.g., Salazar and Salazar, 1996, Bringolf et al., 2007) despite the complexity of the unionid life cycle. After fertilization through sperm-broadcasting, female unionids will mature broods in their marsupial gills. After maturation, the obligate parasite larvae, glochidia, are released into the water column (Matteson, 1955, Waller and Lasee, 1997). Glochidia will then attach to the gills of a host, usually a fish (Haag, 2012), and form a cyst where metamorphosis will take place. The larvae receive nutrients from the host tissue as they differentiate into metamorphosed juveniles and fall from the gills of the host fish (Haag et al., 1995). The excystment after metamorphosis is commonly called juvenile drop off. Assessments of the juvenile stage do not account for the vulnerability of glochidia, which have comparable or greater levels of sensitivity to pesticides including atrazine, heavy metals, and ammonia relative to the later juvenile stage (Jacobson et al., 1997, Augspurger et al., 2003, Bringolf et al., 2007, Wang et al., 2007, Bringolf et al., 2010). Furthermore, such partial life cycle tests examining the response of juveniles or free-floating glochidia neglect to consider how transformation success (i.e., the number of glochidia that metamorphose to juveniles and excise) may be impacted. In marine bivalves, which metamorphose without the use of a host fish, metamorphosis is more sensitive to legacy contaminants than other toxicological endpoints assessed for juveniles such as growth and mortality (Zhang, 2002, Wang et al., 2012). In unionids, however, ecotoxicological tests of glochidia typically assess free-floating individuals for a maximum period of 24 h, neglecting the importance of exposure while in brood or while encapsulated within a cyst on the host fish (Cope et al., 2008). A partial life cycle test that encompasses glochidia in brood, during encystment and the subsequent period of encapsulation, and successful transformation is needed to determine how chemical exposures in each life stage could lead to population level impacts. Movement towards toxicity tests on fitness related parameters such as transformation success is crucial to assess impacts on populations rather than traditional tests of lethality that assess organismal impacts.

At least two components of transformation can be shaped by the presence of environmental stress including: (1) how many glochidia successfully metamorphose and (2) the duration of metamorphosis. Reduced juvenile cohorts alter the population. The probability of juvenile unionids surviving to adulthood transition to adulthood range from 15 to 68% (Neves and Widlak, 1987, Haag and Staton, 2003, Hanlon and Neves, 2006). Estimates of juvenile survival are even lower for those metamorphosed earlier or later in the season than anticipated (Hanlon and Neves, 2006). Thus, if CECs alter the duration of transformation as seen for other stressors (Roberts and Barnhart, 1999), it could lead to population level consequences that may be detrimental to unionid conservation regardless of if the same proportion of juveniles transform.

We investigated the impacts of an ecologically relevant mixture of CECs found in the Laurentian Great Lakes basin on both the success and duration of unionid metamorphosis and used the generated empirical data to simulate populations under CEC stress. We used two CEC mixtures, based on large scale surveys of the basin, to mimic the differing combinations of CECs found in agricultural and urban environments (Baldwin et al., 2016, Elliott et al., 2018, Cipoletti et al., 2019). The common unionid species, *Lampsilis cardium* (Plain Pocketbook), and artificially infested host fish, *Micropterus salmoides* (Largemouth Bass), were exposed to CEC mixtures for either a short- or long-term duration during July through October 2017 and 2018 to explore the interactions between CEC concentrations and exposure time. First, the exposure study allowed us to address three questions: (1) Does duration of host fish and unionid exposure prior to infestation affect transformation? (2) Do CEC mixtures alter duration of encapsulation of unionid larvae on host fish? and (3) Do CEC mixtures alter the total number of transformed unionid juveniles that drop off? Then, using a combination of our empirically derived data and previous studies on unionid life history, we constructed Lefkovitch matrix characterizing crucial unionid life stages to address a question of conservation interest: Can CEC exposure lead to population-level consequences in unionids?

## Methods

### Animal collection and housing

The Central Michigan University vivarium facility (Mount Pleasant, Michigan, USA 48859) housed *Lampsilis cardium* of adult size and a minimum of 3 years of age (length = 89.9 ± 12.8 mm) and two-year-old host fish *Micropterus salmoides* (total length = 97.9 ± 25.4 mm, mass = 12.1 ± 10.4 g). We hand-collected gravid female unionids from the Grand River at Lyons, MI (42.985731˚ N, -84.945412˚ W; Michigan Cultural or Scientific Collection and Threatened and Endangered Species Permit (2017–2018) and US Fish and Wildlife Services Federal Endangered Species Permit (TE71821A-3)). *Lampsilis cardium* was selected for the study as *L. cardium* is a ubiquitous and abundant species of unionid in North America that is not threatened (global conservation status: least concern; Bogan et al., 2017), is found in ample numbers at the collection site (Woolnough and Barnett, 2013), and live in unionid communities with rare species (Woolnough and Barnett, 2013). Stoney Creek Fisheries and Equipment (Grant, Michigan, USA 49327) supplied host fish for both short- and long-term exposures. Animals were maintained and acclimated to standardized laboratory conditions for two weeks prior to experiments.

CEC mixtures were developed by the US Geological Survey and US Fish and Wildlife Service based on widespread surveys of the Great Lakes basin (Baldwin et al., 2016, Choy et al., 2017, Elliott et al., 2017, Elliott et al., 2018) and represented common co-occuring CEC mixtures (Table 1) from areas dominated by agriculture or urban developed land use (Elliott et al., 2018). Treatments (Table 1) consisted of urban low (UL), medium (UM), and high (UH); agricultural low (AL), medium (AM), and high (AH); a water control (CW); and an ethanol control (CE). Medium concentrations reflect maximum CEC levels most commonly detected in nature (i.e., ecologically relevant); low and high concentrations reflect a tenfold decrease and increase respectively (Table 1; Baldwin et al., 2016, Choy et al., 2017, Elliott et al., 2017, Elliott et al., 2018). However, we must note that nominal concentrations were not achieved for most treatments, and some contamination occurred. Unknown factors associated with analyzing emerging contaminants including limitation in chemical quantification (e.g., elevated detection limits due to evolving extraction methods, and limits in instrumentation), alterations in chemical and handling methods leading to contaminant aerosolization, and unforeseen chemical/biological interactions (e.g., chemical instability, uptake by unionid/algae, and aquaria tubing among others) are likely causes (Richardson and Ternes, 2014). Regardless, the exposure was a gradient of contamination that reflects realistic chemical mixtures found in the environment and highlights the challenges of working with and quantifying CECs. A solvent control (CE, 0.5 µL EtOH/L; below ASTM International 2013 recommendations of 100 µL EtOH/L) treatment was used to evaluate the potential effects of the solvent carrier in CEC mixtures (ASTM International 2013). The solvent control (CE) contained concentrations of EtOH identical to those in all CEC treatments (i.e., EtOH concentrations were 0.5 µL/L in all CEC treatments). All treatments and controls were implemented in 2017 trials while only controls and medium treatments, selected for ecological relevance, were implemented in 2018 trials due to logistical constraints.

**Table 1:** A summary of measured and nominal concentrations measured in ng/L of primary mixture treatments for the 2017 (Measured 1) and 2018 (Measured 2) exposures as measured by the USGS National Water Quality Laboratory using LC-MS techniques

		Treatments (ng/L)							
Chemical		CW	CE	AL	AM	AH	UL	UM	UH
4-Nonylphenol	Nominal	0	0	18.81	188.1	1881	371	3710	37100
	Measured 1	ND (<100)	ND (<100)	ND (<100)	ND (<100)	ND (<100)	ND (<100)	1846 ± 1464	14240 ± 6823
	Measured 2	335 ± 726	254 ± 686	NA	251 ± 753	NA	NA	927 ± 1947	NA
5-Methyl-1H-	Nominal	0	0	0	0	0	668	6680	66800
benzotriazole	Measured 1	530 ± 1903	338 ± 435	122 ± 126	36 ± 46	308 ± 1039	1020 ± 670	5607 ± 4787	41062 ± 47658
	Measured 2	ND (<10)	ND (<10)	NA	ND (<10)	NA	NA	6320 ± 6111	NA
Atrazine	Nominal	0	0	40	400	4000	0	0	0
	Measured 1	8 ± 11	ND (<10)	97 ± 30	848 ± 160	892 ± 961	ND (<10)	ND (<10)	ND (<10)
	Measured 2	ND (<5)	ND (<5)	NA	1282 ± 216	NA	NA	ND (<5)	NA
Bisphenol A	Nominal	0	0	6	60	600	300	3000	30000
(BPA)	Measured 1	123 ± 269	91 ± 62	ND (<100)	ND (<100)	422 ± 466	198 ± 119	1721 ± 1135	15803 ± 13152
	Measured 2	128 ± 358	322 ± 974	NA	245 ± 706	NA	NA	457 ± 901	NA
Bromacil	Nominal	0	0	12	120	1200	0	0	0
	Measured 1	ND (<50)	ND (<50)	ND (<50)	216 ± 54	2048 ± 451	ND (<50)	ND (<50)	ND (<50)
	Measured 2	ND (<10)	ND (<10)	NA	300 ± 46	NA	NA	ND (<10)	NA
N,N-Diethyl-m-	Nominal	0	0	20	200	2000	160	1600	16000
toluamide (DEET)	Measured 1	96 ± 228	112 ± 117	123 ± 59	432 ± 82	2129 ± 1073	377 ± 167	1931 ± 933	11389 ± 7287
	Measured 2	ND (<20)	ND (<20)	NA	492 ± 142	NA	NA	1292 ± 1310	NA
Desvenlafaxine	Nominal	0	0	0	0	0	58.3	583	5830
	Measured 1	21 ± 48	6 ± 4	ND (<D10)	ND (<10)	24 ± 75	43 ± 21	343 ± 179	2701 ± 2590
	Measured 2	ND (<10)	ND (<10)	NA	ND (<10)	NA	NA	1242 ± 1370	NA
Estrone	Nominal	0	0	2.44	24	244	0.69	6.9	69
	Measured 1	ND (<100)	ND (<100)	ND (<100)	ND (<100)	101 ± 79	ND (<100)	ND (<100)	63 ± 30
	Measured 2	ND (<50)	ND (<50)	NA	ND (<50)	ND (<50)	NA	ND (<50)	NA
Fexofenadine	Nominal	0	0	0	0	0	100	1000	10000
	Measured 1	54 ± 121	71 ± 136	50 ± 105	ND (<20)	77 ± 261	315 ± 88	2311 ± 712	13181 ± 3711
	Measured 2	ND (<40)	ND (<40)	NA	ND (<40)	NA	NA	3268 ± 1059	NA
Hexahydromethylcyclo	Nominal	0	0	0	0	0	218	2180	21800
pentabenzopyran	Measured 1	ND (<100)	ND (<100)	ND (<100)	DN (<100)	ND (<100)	69 ± 49	1019 ± 387	7290 ± 2009
	Measured 2	ND (<40)	ND (<40)	NA	ND (<40)	NA	NA	1614 ± 612	NA
Metformin	Nominal	0	0	0	0	0	121	1210	12100
	Measured 1	336 ± 1108	ND (<10)	143 ± 286	55 ± 173	98 ± 350	228 ± 149	1985 ± 658	9801 ± 3311
	Measured 2	ND (<100)	ND (<100)	NA	ND (<100)	NA	NA	2568 ± 198	NA
Metolachlor	Nominal	0	0	17	170	1700	0	0	0
	Measured 1	ND (<10)	ND (<10)	69 ± 17	372 ± 57	3645 ± 363	6 ± 5	ND (<10)	ND (<10)
	Measured 2	ND (<5)	3 ± 1	NA	487 ± 61	NA	NA	ND (<5)	NA
Sulfamethoxazole	Nominal	0	0	0	0	0	55.9	559	5590
	Measured 1	13 ± 25	11 ± 10	6 ± 3	6 ± 5	14 ± 34	36 ± 17	458 ± 162	5163 ± 1065
	Measured 2	ND (<5)	ND (<5)	NA	ND (<5)	NA	NA	741 ± 111	NA
Tris(2-butoxyethyl)	Nominal	0	0	210	2100	21000	1350	13500	135000
phosphate (TBEP)	Measured 1	727 ± 1924	1863 ± 1526	1253 ± 396	2850 ± 1126	14797 ± 8397	3720 ± 3312	10079 ± 6489	103419 ± 58047
	Measured 2	ND (<50)	ND (<50)	NA	2021 ± 1391	NA	NA	6940 ± 9238	NA

Measured concentrations were calculated by averaging the concentrations (± standard deviation) of 16 biweekly water samples (2017) and 15 biweekly water samples (2018) where one-half the median detection limit was substituted for reads below the detection limit. *ND* non detects (detection limit in parentheses), *NA* not assessed due to study design

Each CEC treatment and control used a closed static-renewal AquaHabitat™ system. Short-term exposures lasted 40 days. Each system housed five females in a 10 L tank for short-term exposures (n_female *L. cardium*/treatment_ = 5). Long-term exposures lasted 100 days. For each treatment of the long-term exposure, five female unionids were housed in a 10 L tank and an additional five females were housed in individual 3 L tanks with a male pair for a concurrent study (n_female *L. cardium*/treatment_ = 10; concurrent study: Richard et al. in prep). Standard protocols for an exposure study of these parts of the unionid life cycle are not available, but, for glochidia and juvenile exposure studies, a minimum of 3 females are required (ASTM International 2013). We fed unionids a minimum of 0.5 mL per individual (i.e., 1 mL per 3 L tank, 3 mL per 10 L tank) twice a week using a mixture of *Nannochloropsis* spp. and Shellfish diet (Reed Mariculture) to mimic excess diet used in other standardized studies while not creating a scenario of algal buildup on tanks (Wang et al., 2007). Three liter tanks for short- and long-term exposures housed individual *M. salmoides* (n_long-term/treatment_ = ~20, n_short-term/treatment_ = ~25). Host fish were also fed twice per week; each fish was given a minimum of five food items from a mixture consisting of frozen Hikari Bio-Pure brine shrimp and bloodworms from (Hikari Sales USA Inc., Hayward, CA 94545) dissolved in dechlorinated H_2_O.

Water was changed three to four times a day with addition of primary mixtures to ensure consistent levels of contaminants and eliminate possible confounding factors such as ammonia build up (ASTM International 2013). We added new, dechlorinated water to holding tanks of each system in excess of the total system volume at a rate similar to that of system flow, allowing water returning to the holding tank to flow out of the system as clean water began circulation. This method reduced stress on the animals and eliminated animal handling. Prior to infestations, four water changes per day were completed, the number of water changes was reduced to three per day for the remainder of exposures based on acceptable water quality parameters (e.g., no evidence of ammonia build-up or pH changes between water changes) and CEC concentrations in preliminary data. Water quality parameters (pH, total chlorine levels (mg/L), free chlorine levels (mg/L), dissolved oxygen (percent and mg/L), ammonia (ppm), temperature (°C)) were recorded twice per week to assure consistent conditions; temperature was monitored to ensure temperatures remained between 18–21 °C (SI Table 1, ASTM International 2013). Laboratory experiments followed protocol approved by Central Michigan University Institutional Animal Care and Use Committee (IACUC) under IACUC approval number 17-11.

### Infestation

After 12 days of exposure for the short-term study or 60 days of exposure for the long-term study, *M. salmoides* were infested with glochidia from gravid female *L. cardium* of the same treatment and same exposure duration using standard procedures (Zale and Neves, 1982; Yeager and Neves, 1986; Yeager and Saylor, 1995). We used DI H_2_O in a 23-gauge syringe to nonlethally flush glochidia from the unionids’ marsupial gills. Flushed material was noted as containing eggs, glochidia, or a mix using a Leica EZ4W stereomicroscope. If glochidia were present, a viability test was performed wherein a drop of fully saturated saline solution was added to approximately 100 counted glochidia, and the number of glochidia that closed in response to the saline solution were considered viable. Glochidia viability was high (>75%) for all females, and thus all gravid unionids were used. A single female’s glochidia was then divided into gridded petri dishes; each petri dish was assigned to an individual *M. salmoides* within treatment to allow correlations between the number of transformed juveniles and female glochidia donor. No individual *L. cardium* exceeded a total of ten host fish infestations to maintain high densities and variability of glochidia source throughout treatments. We quantified the number of glochidia in at least three 1 cm^2^ grids for each dish except when density exceeded 250 glochidia/cm^2^. Grids above 250 glochidia/cm^2^ was denoted as >250 to avoid over or under estimations by counts of dense areas where individual glochidia may not be completely visible due to overlapping. Glochidia concentrations exceeded concentrations known to fully saturate the gills of *M. salmoides* (Dodd et al., 2005). Air stones provided oxygenation to *M. salmoides* tanks, and water circulation from the tank system was turned off, during this initial glochidia addition, to prevent glochidia from being washed through the outflow standpipe at the head of individual tanks into general water circulation. Tanks remained disconnected from water circulation for approximately 45 min to allow glochidia to attach to the gills or drop to the bottom of the tanks. PVC pipe sections covered with 118 µm mesh (smaller than size of glochidia), hereafter called filters, were attached to tank outflows to collect any glochidia or transformed juveniles as they dropped off the gills of *M. salmoides*, and system flow was restored. We removed and counted filters every other day from one day after infestation to the end final day of exposure. We quantified the number of glochidia, fully metamorphosed juveniles (based on the presence of a shell growth line, foot, and formation of a gut), and partially metamorphosed juveniles (only one or two of the above criteria were met; Yeager and Saylor 1995).

### Statistical analysis

A variety of modelling techniques have been used to describe the presence and the population dynamics of unionids (e.g., Villella et al., 2004, Newton et al., 2008, Inoue et al., 2014), but few have applied multivariate modelling techniques to the process of transformation (Tremblay et al., 2016, Marwaha et al., 2017). In many cases, transformation success is only described by a percent transformation where the number of transformed juveniles is divided by the number of glochidia estimated to have attached to the fish (Dodd et al., 2005, Tremblay et al., 2016, Caldwell et al., 2016). Such percentage calculations may be inaccurate and mischaracterize transformation success if the number of glochidia attached is not accurately estimated. Estimating attached glochidia is difficult as host fish are typically inoculated by high numbers of glochidia wherein researchers are unable to count the initial addition accurately; furthermore, glochidia that do not attach may degrade in tanks and be mischaracterized as attached. We therefore used two modelling techniques to assess juvenile transformation with increased precision as described below.

### Zero-inflated Poisson general linearized mixed model (ZIP-GLMM)

The first method used a zero-inflated Poisson GLMM (ZIP-GLMM; package: glmADMB; Bolker et al., 2012). Previously, the GLMM technique has been used to describe transformation success of an individual glochidia as a binary response (Marwaha et al., 2017), but here, we used GLMMs to determine the number of juveniles dropping off a host fish each day throughout the transformation period. The selected distribution allowed for the use of juvenile count data and adjusted for the zero-inflation during initial days after infestation (Min and Agresti, 2005, Bolker et al., 2012, Harrison, 2014). The dataset was truncated to the first 27 days after infestation to allow comparison between exposure durations (i.e., the short- and long-term trials). Data was sourced from both 2017 and 2018 trials (Table 2). Our model assumes peak drop off, under vivarium conditions, would occur on or around 27 days (Watters, 1997, Gibson, 2014). Each unique host fish and the *L. cardium* from which glochidia originated were random effects used in all models to account for repeated measures and individual deviations from the population estimated means (Min and Agresti, 2005, Bolker et al., 2012). Fixed effects included group (control for CE and CW, agricultural for AL, AM, and AH, or urban for UL, UM, and UH), treatment (CE, CW, AL, AM, AH, UL, UM, UH), days since the fish was infested, days the fish had been exposed to the stressors, and interactions between the group or treatment variable and the time variables. To avoid rank deficiency, in which one variable is a nested subset of another, group and treatment were not included in the same model. No concentration models used only time data (days since infestation or days since exposure) to assess the improvement of models by including CEC data. We compared candidate models using Akaike Information Criterion corrected for sample size (AICc). Models were constructed from a random 75% subsample of the data (training data) with 25% excluded for model validation. Competing models were tested and compared using AICc scores (package: MuMIn; Barton, 2018) and model weight (Table 3). The R^2^ value assessed fit of the model (package: MuMIn; Barton, 2018); although interpretation of R^2^ can be challenging with ecological data (Møller and Jennions, 2002), we considered R^2^ values of 0.18–0.51 to be a moderately explain the variation of a model (Plonsky and Ghanbar, 2018).

**Table 2:** Sample sizes of host fish and unionids used in ZIP-GLMM modelling.

		Short-term		Long-term	
Treatment	Year	*M. salmoides*	*L. cardium*	*M. salmoides*	*L. cardium*
CW	2017	20	4	0	0
	2018	15	4	10	2
CE	2017	20	4	0	0
	2018	13	4	11	1
AL	2017	20	2	0	0
	2018	–	–	–	–
AM	2017	12	3	13	2
	2018	14	3	10	1
AH	2017	18	2	5	1
	2018	–	–	–	–
UL	2017	10	2	5	1
	2018	–	–	–	–
UM	2017	12	3	10	2
	2018	14	5	10	1
UH	2017	0	0	20	4
	2018	–	–	–	–

Treatment codes are used for control water (CW), control ethanol (CE), agricultural low, medium and high (AL, AM, AH respectively), and urban low, medium, and high (UL, UM, UH respectively). Dashes represent when exposures were not performed and thus not included in models. Zeros represent when exposures were performed but high mortality prevented sufficient sample size for use in models

Number of *Micropterus salmoides* host fish and *Lampsilis cardium* unionids used per treatment and exposure type each year varied.

**Table 3:** Summary of convergent ZIP-GLMM models of *Lampsilis cardium* juvenile transformation on *Micropterus salmoides* host fish with improved performance compared to best fit model excluding CEC treatment

Model name	Group	Treatment	Days since infestation	Days of exposure	Group: Days of exposure	Group: Days since infestation	Treatment: Days of exposure	Treatment: Days since infestation	∆AICc
Treatment.6		x	x					x	0
Treatment.2		x	x	x				x	3.6
Group.1	x		x	x	x				80.3
Group.2	x		x	x		x			87.2
Treatment.1.2		x	x	x					119.6
Treatment.3.6		x	x						123.9
Treatment.5		x		x					132.0
No.concentration.1			x	x					175.0
Null									339.3

Group variable refers to control, agricultural, or urban treatments regardless of concentration (e.g., AL, AM, or AH would simply be classified as agricultural), and the treatment variable refers to both group and specific concentration (i.e., CW, CE, AL, AM, AH, UL, UM, and UH). Models were named for their inclusion of Group or Treatment variables followed by a number indicating the combination of variables and interactions included. Days since infestation represents the number of days past infestation in which the day of infestation was considered day 0; days of exposure represents the number of days the *M. salmoides* had been in treatment conditions where day 0 was the initial day animals were moved into individual tanks. Models were assessed using their ∆AIC score adjusted for small sample size and named based on their score

### Response curves

The second method to describe *L. cardium* drop off from *M. salmoides* included data from only the long-term exposures of both trials, 2017 and 2018, to allow description of drop off beyond the 27 day period described in the ZIP-GLMM. Response curves may be constructed by fitting a variety of polynomial or spline shapes to describe the biological response of an individual or group of individuals over a variable, such as time (Greenland, 1995, Kreutzweiser et al., 2004). Response curves have been previously applied to describe metamorphosis of both aquatic vertebrate and invertebrate larvae (Hota and Dash, 1981, Burke, 1984). To describe the full two to three week period of metamorphosis (Watters, 1997, Gibson, 2014), only fish from the long-term exposures that survived for a minimum of 30 days were included in the analysis (*n* = 84). The AL treatment was excluded from this analysis due to high mortality rates of fish and low gravidity of *L. cardium* during the 2017 trials. MATLAB software was used to visualize and select the best approximate curve to fit the data using the curve fitting tool for a subsample of the fish; a third order polynomial was selected based on the highest R^2^ score compared to other fits including Gaussian, Weibull, linear, first and second order polynomials, and sum of sine distributions. All fish were then fit for individual curves with intercepts set at zero using base R software. Critical points (i.e., where the slope of the fitted curve is equal to zero) were calculated for each fish based on the derivative of the curve. We compared critical points among treatments to determine changes in both the day of highest drop off (the x-coordinate of the critical point) and number of juveniles excising at this peak day (the y-coordinate of the critical point; i.e., the number of juveniles that would fall off in a two-day period given filter counts for juveniles were completed every other day in this study). We used an analysis of variance (ANOVA) with adjustment for unequal sample size followed by *post-hoc* Tukey’s comparisons (Zar, 2010) to compare critical point coordinates. If assumptions of the ANOVA were not met, we used the nonparametric equivalent, a Kruskal-Wallis test, followed by a Dunn’s test to consider pairwise differences between treatments (Zar, 2010). We omitted curves of *M. salmoides* that did not result in transformed juveniles for comparisons of peak day as a critical point on the x-axis (time) could not be calculated; curves associated with fish that did not result in transformed juveniles were included in comparisons of juveniles transformed, however, as zero production of juveniles could indicate a true effect of the treatment.

### Lefkovitch matrix

**Fig. 1:**
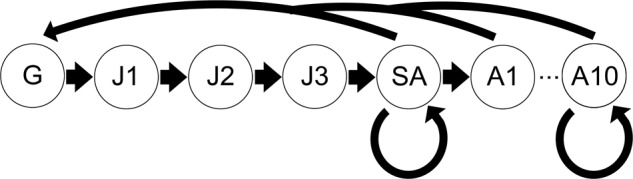
Stage-based Lefkovitch model representing key transitions in the unionid life span. Parameter values can be found in Table 4

A stage-based Lefkovich matrix model was constructed based on the unionid literature to describe transitions from glochidia to juvenile, juvenile to subadult, and subadult through adult stages (Fig. 1, Table 4). The model allows population projections of a single sex (females) to inform conservation (Crouse et al., 1987). Similar age-structured population models have been used to assess the likelihood of unionid reintroduction success (Jones et al., 2012). The life stages include glochidia, three juvenile stages, one subadult stage, and ten adult stages. The early years of life are critical time points wherein juveniles differ in survivorship from subadults or adults (Haag et al., 2019, Bauer, 2001). Survivorship and fecundity are related to size structure prior to reaching an asymptote (Haag and Staton, 2003); thus, ten adult stages were included with appropriate scaling to fecundity and survivorship in addition to the subadult stage where reproduction is possible but fecundity is comparatively much lower (Haag and Staton, 2003). The transition between glochidia to juvenile (transformation) was scaled to reflect CEC exposures using empirical data. The total numbers of juveniles transformed as predicted by models were averaged within treatment for both ZIP-GLMM and response curve models. Using model-based predictions avoids confounding issues such as missing data points (e.g., if a tank was not counted for one temporal point or experienced mortality before the completion of a transformation curve) and uneven sampling. We reduced the transition between glochidia and juvenile stages by multiplying the ratio of total juveniles transformed in the CEC condition to the total juveniles transformed in CW within each model type and averaged the two ratios. To account for variation among curves, we calculated the standard deviation for each percent change. We then averaged the mean percent change and standard deviations between models to create a single percent change and standard deviation per treatment except for AL for which only ZIP-GLMM calculations were completed. We then used the averaged values to scale lower and upper bounds of uncertainty in our projections by subtracting (lower bound) and adding (upper bound) the standard deviations We thus created three predictions for each treatment, except for controls that were used for scaling and AL; we projected the based on the average percent change, the lowest expected percent change (based on standard deviation), and the highest expected percent change (based on standard deviation). Projections were carried out for 30 years (R package: popbio; Stubben and Milligan, 2007) using population data estimated on size class surveys completed by Newton et al. (2011). We compared the annual population growth rate, the lambda value, among CEC simulations where a lambda (annual population growth rate) above one indicates population increase and below one indicates population decrease. We compared elasticities and sensitivities to determine key life stage transitions under these scenarios.

**Table 4:** Parameters used in Lefkovich matrix found in the literature for the unionid lifecycle including glochidia (G), juvenile (J), subadult (SA) and adult stages (A)

Parameter type	Transition	Value	Species	Source
Survival	G to J1	0.0056	Various unionids	Jansen and Hanson 1991; Bauer 2001
	J1 to J2 J2 to J3	0.5	Various unionids	Haag et al. 2019; Bauer 2001
	SA to SA	0.6	*Lampsilis ovata*	Haag and Staton 2003 (based on reproduction age of *L. cardium*)
	SA to A1	0.2	Various unionids	Haag 2013
	A1 to A2 … A9 to A10	0.8	*Lampsilis siliquoidea* *Lampsilis cariosa* *Amblema plicata*	Anthony et al. 2001; Villella et al. 2004; Hart et al. 2004
Fecundity	SA on G	100 000	*L. ovata*	Haag and Staton 2003
	A1 on G … A10 on G	600 000	*L. ovata* Various unionids	Haag and Staton 2003; Haag 2013

Parameters were taken from closely related species if data from *Lampsilis cardium* were unavailable. Parameters were used in population projections with the exception of the G to J1 (glochidia to juvenile) transition which was scaled for CEC scenarios by empirical data from exposure studies

All analyses used R version 3.5.1 software unless otherwise noted. Figures were constructed using the ggplot2 package (Wickham et al., 2018).

## Results

### ZIP-GLMM

A total of 262 *M. salmoides* used in the analysis were infested with glochidia from 51 female *L. cardium*, resulting in 3590 counts of juvenile transformation (short-term = 2411; long-term = 1179) across the 27 day period were used for modelling of transformation. Training data used to construct models included 2692 counts of filter contents, and 898 counts of filter contents were used for model validation. Number of host fish *M. salmoides* and female *L. cardium* representing each treatment and exposure duration varied with the highest numbers being present in the medium and control treatments as a result of repetition in 2018 trials (Table 2). It is important to note that number of *M. salmoides* varied over time due to host fish mortality. Host fish experiencing mortality did not appear to differ from healthy *M. salmoides* in juveniles transformed based on inspection of these data and model selection.

We selected as the best fit ZIP-GLMM based on AICc score (Table 3). The best-fit model (named Treatment.6) was found to have moderate fit (R^2^ = 0.287) based on predicted results using population averaged random effects (summary of model: Table 5, SI Fig. 1). This may be attributable to low and uneven sample size increasing the variability of the dataset, but low R^2^ values are common in ecological datasets (Møller and Jennions 2002). Random effects were close to zero for both the female *L. cardium* from which glochidia were collected (0.0347 ± 0.0563) and individual host fish (0.0144 ± 0.0352). Significant interaction effects (*p* < 0.05) between treatment and days since infestation occurred for all agricultural treatments (AL, AM, and AH) as well as UM. Estimates of the interaction were positive for agricultural treatments and CW, but the coefficient was negative for UM. Coefficients for treatment effect, however, were all negative with the exception of CE and UM (Table 6, SI Fig. 1).

**Table 5:** Summary of best fit ZIP-GLMM (Treatment.6 in Table 3) describing *Lampsilis cardium* juvenile drop off from *Micropterus salmoides*

		ZIP-GLMM Coefficients	
Treatment	*n*	Treatment ± SE	Treatment: Days since infestation ± SE
CW	45	–	0.13 ± 0.0066
CE	44	0.56 ± 0.47	0.023 ± 0.019
AL	20	−0.43 ± 0.61	0.064 ± 0.017
AM	49	−1.29 ± 0.47	0.13 ± 0.017
AH	23	−3.60 ± 0.99	0.16 ± 0.043
UL	15	−2.31 ± 1.07	0.003 ± 0.059
UM	46	1.25 ± 0.44	−0.052 ± 0.017
UH	14	−251 ± 43100	9.64 ± 166

ZIP-GLMM coefficients (Treatment and Treatment:Days since infestation) were estimated for included variables where CW (control water) was considered to be the base model for which other treatments added additional intercepts. Treatment codes are used for control water (CW), control ethanol (CE), agricultural low, medium and high (AL, AM, AH respectively), and urban low, medium, and high (UL, UM, UH respectively). *SE* standard error

**Table 6:** Comparison of critical points of time response curves fit for *Lampsilis cardium* juvenile transformation on each individual *Micropterus salmoides* host fish using a third order polynomial with a fixed intercept at zero

Treatment	*n*	Max juvenile/fish ± SE	Dunn’s test			Peak day ± SE	Tukey’s test		*n*	Peak day w/o zero slope fish ± SE	Dunn’s test
CW	4	4.71 ± 1.47		ab	24.19 ± 2.17			ab	4	24.19 ± 2.17	bcd
CE	8	0.39 ± 0.16		ac	17.37 ± 3.82			ab	6	23.16 ± 0.67	cd
AM	21	3.16 ± 0.47		ab	28.46 ± 2.52			a	21	28.46 ± 2.52	abcd
AH	5	0.74 ± 0.075		a	31.78 ± 2.20			a	5	31.78 ± 2.20	abc
UL	5	0.029 ± 0.029		cd	5.88 ± 5.88			b	1	29.39	abcd
UM	17	0.51 ± 0.012		ac	23.58 ± 2.42			ab	15	26.72 ± 1.25	abcd
UH	20	0.039 ± 0.017		cd	7.64 ± 3.07			b	5	30.56 ± 2.06	abcd

The average of the critical points for all host fish in a treatment were used to represent the date at which maximum *L. cardium* juvenile production would occur denoted as “peak day”; the highest number of *L. cardium* juveniles dropping off a host fish on a given day denoted as “max juvenile” were estimated using each host fish’s individual curve value at the individual host fish’s critical point. Both calculations of peak day including and excluding zero slope host fish are shown below to demonstrate the need to exclude zero slope host fish in representing the true nature of the system. Visualizations of the data are available in Appendix F. *SE* standard error

### Time response curves

**Fig. 2:**
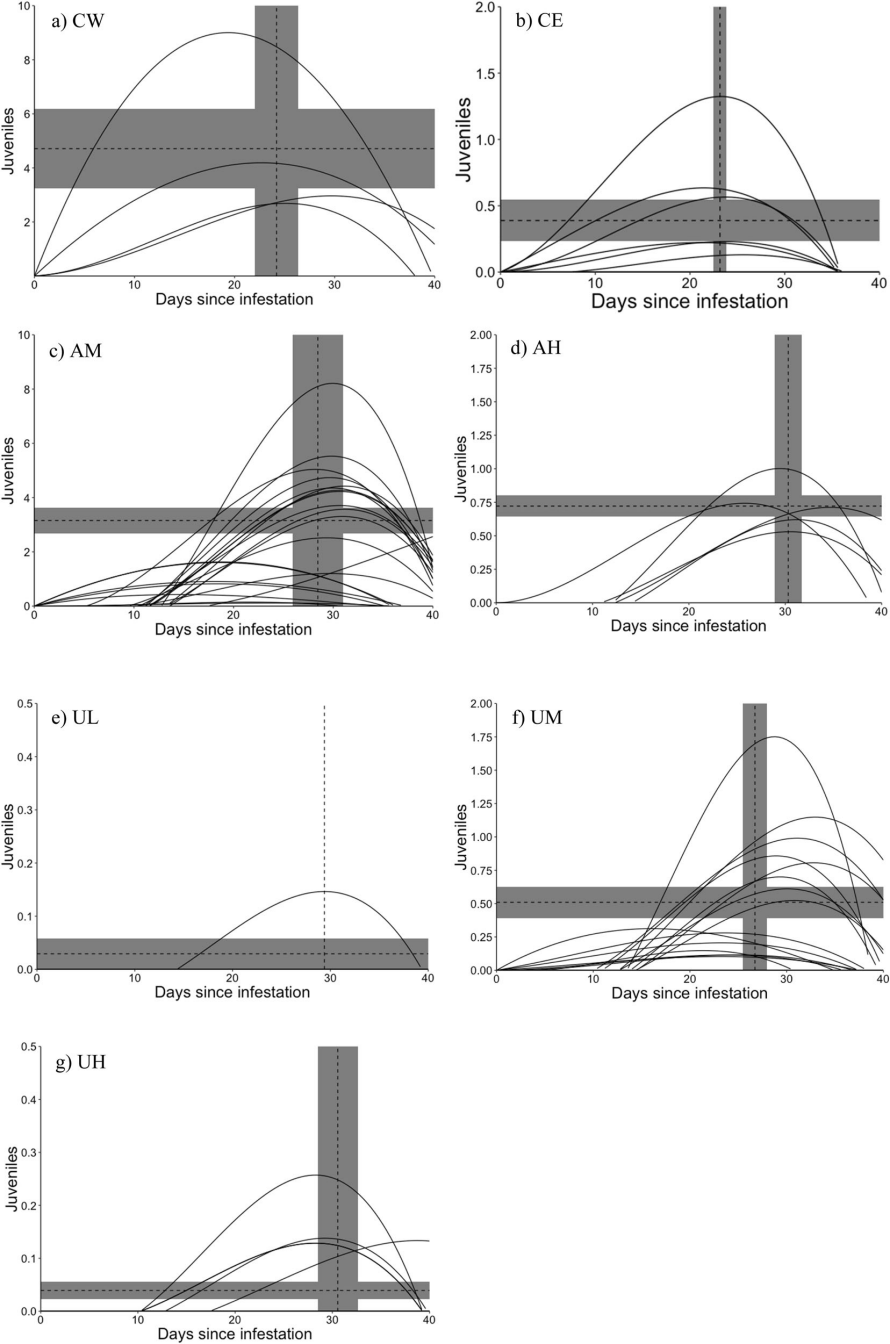
Curves describing transformed *Lampsilis cardium* juvenile drop off following a third order polynomial fit for each *Micropterus salmoides*. Exposures are defined as controls using water (CW; **a**) or ethanol (CE; **b**), agricultural contaminants mixtures at medium (AM; **c**) or high concentrations (AH; **d**), or urban contaminant mixtures at a low (UL; **e**), medium (UM; **f**), or high (UH; **g**) concentration. Dashed vertical lines represent the average day of peak drop of for the treatment, not including zero slope host fish while dashed horizontal lines represent the average number of *L. cardium* juveniles dropping off per host fish at the peak day; surrounding grey boxes represent standard error. Note that y-axis is on different scales for each treatment. In AM and UM, two sets of curves are present; lower curves were generally from the 2018 trials though differences between year were not significant (*p* < 0.05). Sample sizes are summarized in Table 2 (*L. cardium*) and 5 (*M. salmoides*)

**Fig. 3:**
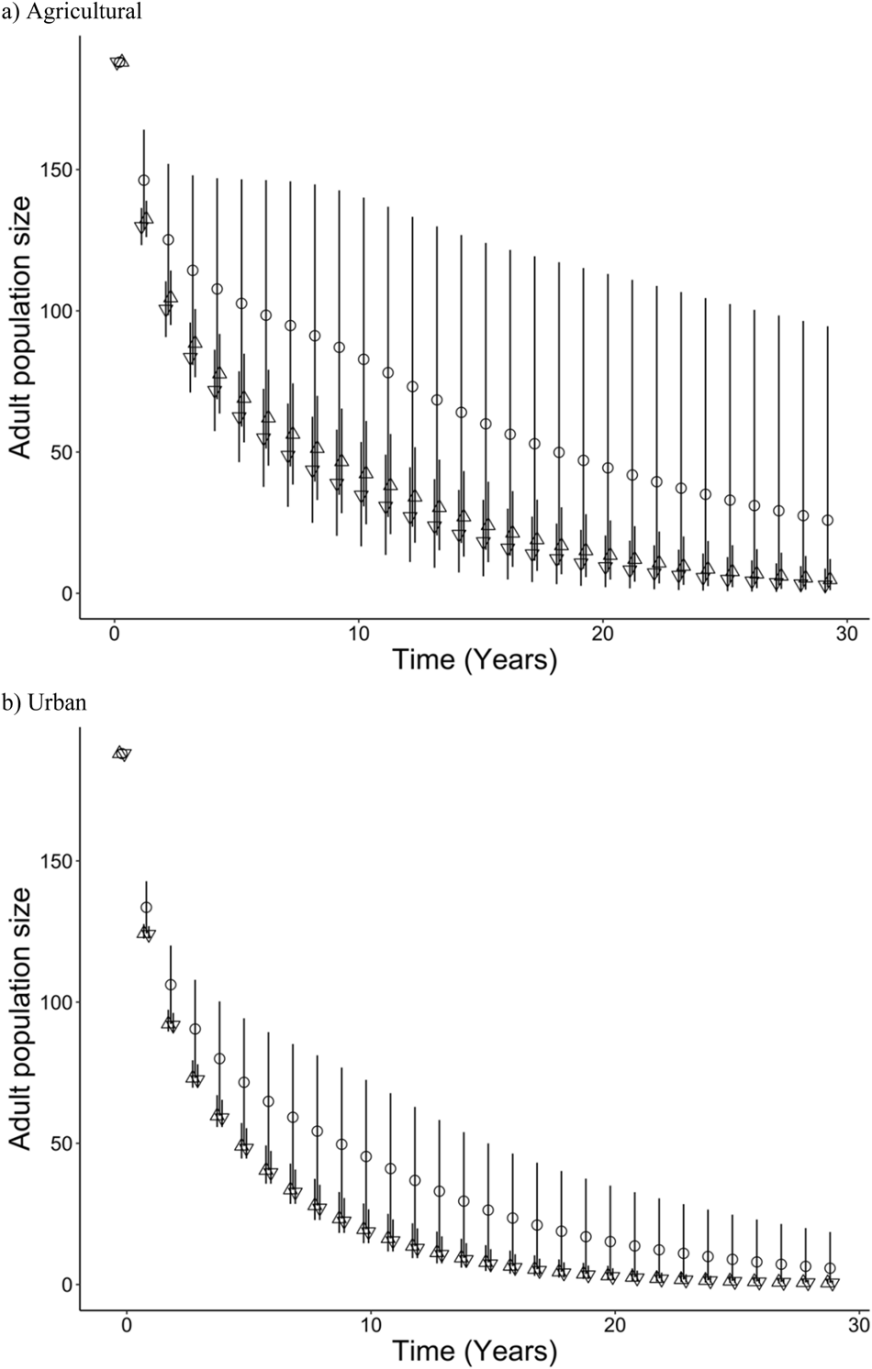
Population predictions for adult population sizes of *Lampsilis cardium* under CEC exposure conditions. Exposures are defined as agricultural contaminants mixtures (**a**) at medium (**a**; circles), high (**a**; triangles pointing up), or low (**a**; triangles pointing down) concentrations as well as urban contaminant mixtures (**b**) at a medium (**b**, circles), high (**b**; triangles pointing up), and low (**b**; triangles pointing down). Error bars represented the highest and lowest ratios of treatment to control transformation conditions in empirical work

Metamorphosis data from 80 host fish fit third order polynomial models to assess the day of peak drop off and peak juvenile production. The peak day of drop off (i.e., the x-coordinate of the critical point of time response curves, Fig. 2), including fish who did not produce juveniles, was estimated as close to zero for some treatments and did not accurately reflect scenarios in which excystment did occur (Table 6). When data with day zero estimates for drop off were excluded, peak day of juvenile drop off differed among treatments in a way that may be ecologically meaningful though not significant (*p* = 0.16, Table 6). *Micropterus salmoides* exposed to CECs, both agricultural and urban mixtures, tended to have a later date of peak drop off compared to host fish in control treatments (Table 6, Fig. 2). Maximum juvenile production (i.e., the y-coordinate of the critical point of time response curves representing the day the most juveniles would drop off, Fig. 2) differed among treatments (*p* « 0.05). CW and AM treatments were predicted to result in similar numbers of juveniles and have the highest drop off rates while UL and UH were similar to one another with low predicted numbers of juveniles (Table 6, Fig. 3).

### Lefkovitch matrix

**Fig. 4:**
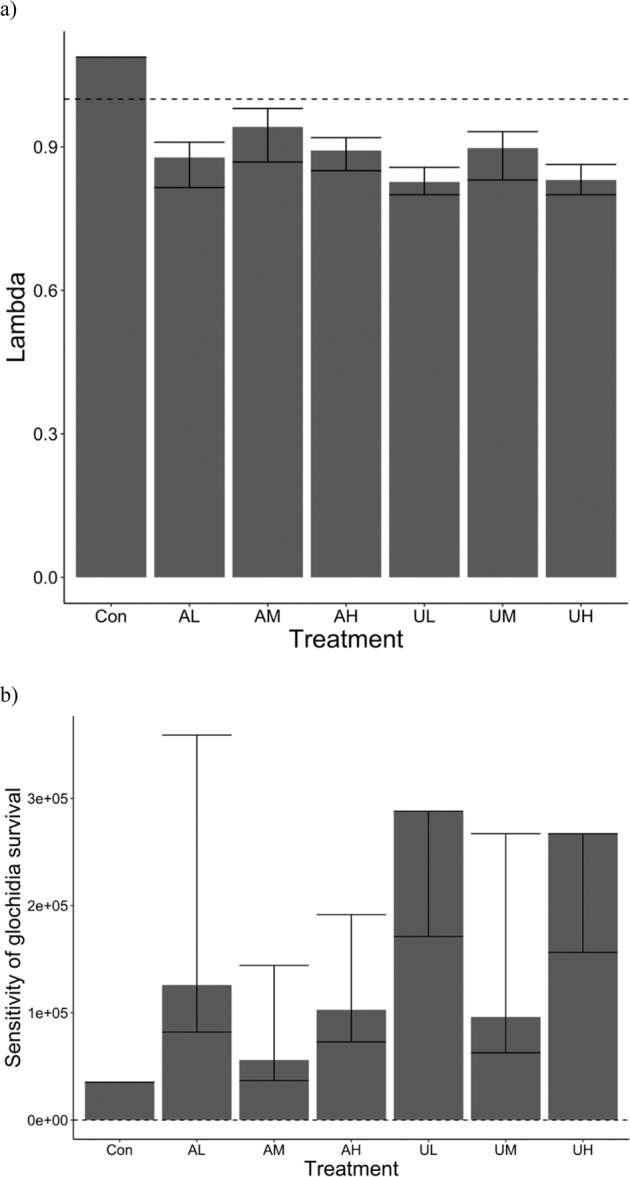
Population growth rate and sensitivity of glochidia survival and metamorphosis of *Lampsilis cardium* predictions after chemical exposures. Annual population growth rate (lambda, **a**) and sensitivity of glochidia survival and metamorphosis (**b**) of Lefkovich models under CEC exposure conditions. Error bars represented the highest and lowest ratios of treatment to control transformation conditions in empirical work. The dashed line at one on panel **a** represents the threshold for population growth. Sensitivity analysis could not be completed for the upper bound of UL and the upper bound of UH for **b** and thus are excluded

Lefkovitch matrices predicted local extirpation of *L. cardium* under all CEC exposure conditions within the 30-year prediction period (Fig. 3). We noted that percent reductions were predicted to be stronger in ZIP-GLMM by approximately 5% compared to time response curve estimations. We believe that the average of the two therefore represents a semi-conservative measure of metamorphic reduction to reduce type I error. Lambda values, the annual population growth rates, were below one for the majority of conditions with the exception of controls, indicating population decline (Fig. 4a). Under all conditions, including control, the survival of glochidia to the juvenile stage had the highest sensitivity (Fig. 4b) and adult stage fecundity had the highest elasticity. It must be noted that matrix structure and near zero values prevented eigen analysis for the lower bound of UL and the upper bound of UH. Thus, sensitivities are only known for the average values and one bound for UL and UH.

## Discussion

Our empirical data demonstrated a clear reduction in unionid juvenile transformation during contaminant mixture stress that was then projected to lead to unionid population extirpation. To our knowledge, this is one of the first studies to assess the effects of chemical stress on the full transformation process, including maternal, host fish, and glochidia exposures. CEC exposure impacted both the duration of encapsulation and the number of *L. cardium* juveniles that transformed (Table 6) though there was not a difference in exposure durations assessed (Table 3). Furthermore, Lefkovitch stage-based modelling predicts that the impact to transformation, the most sensitive life-stage transition, may result in severe population declines within a 30-year period. Consistent results among CEC treatments indicate that declines are possible in areas impacted by ecologically relevant agricultural or urban associated contaminants.

Our study indicates CEC mixture stressors reduced recruitment causing overall population declines, yet the physiological cascade of events that leads to this loss is unknown. Modes of action have not been resolved for many chemical classes in unionids and may differ due to unique physiology as seen for endocrine disrupting contaminants to which unionids do not show expected responses due to a lack of analogous receptors (Newton and Cope, 2007). Rather than seeking modes of action for each contaminant individually, we were able to address how realistic combinations of CECs negatively impact recruitment as CECs tend to occur in complex mixtures rather than in isolation (Elliot et al., 2017). Although we do not address the potential for chemical interactions among the complex mixtures used in this study, we see considering these interactions as a fruitful future avenue of research to address to further consider nuanced rational for responses observed in this study. This study provides an important first step by validating complex CEC mixtures with varying unknown modes of action for mussels do limit reproduction success through altered transformation duration and rates. Future research designed to understand the mechanisms of these alterations are needed for evaluating the extent of CEC impacts and to determine which chemical classes most influence these deleterious effects.

The two exposure durations in this study resulted in similar impacts on unionid juvenile transformation success. Previous guidelines have not established an appropriate duration of contaminant exposures for host fish transformation studies (ASTM International, 2013). Many studies involving glochidia have quantified effects of short exposures on free-floating larvae not encysted on a host fish (Keller and Ruessler, 1997, Keller and Augspurger, 2005) and have thus neglected the question of whether maternal or host exposure can influence transformation success. It is unclear if the unionid marsupial gill structure may have precluded glochidia exposure in brood prior to infestation. While glochidia viability was above 80% for all treatments prior to infestation, fitness of the glochidia could not be fully assessed and therefore impacts of in vivo exposure is unknown. The gills separate glochidia from respiratory processes that would permit contact with contaminated water (Jacobson et al., 1997, Cope et al., 2008) while still allowing for selective ion (nutrient) exchange between the mother and offspring (Schwartz and Dimock, 2001), though transport of organic contaminants, such as used in this study, have not been assessed. Mechanisms of glochidial exposure during brooding are limited (e.g., Pynnönen, 1995), especially the consideration of how gills may protect or mediate exposure, and merit further investigation.

After release from the maternal brood, glochidia experienced both a brief direct exposure to contaminants during host fish infestation and potential indirect exposure while encysted on host fish. Few studies address the importance of host fish contaminant exposure relative to glochidia exposure in determining transformation rates, but available evidence indicates glochidia exposure as a more important factor (Jacobson et al., 1997, Hazelton et al., 2012, Beggel and Geist, 2015, Gillis et al., 2021). Maternal unionids, glochidia, and host fish were from the same exposure duration treatments, and thus we cannot determine if juvenile transformation was influenced by host fish exposure, direct contact of glochidia with CECs during infestation, or previously discussed glochidia exposure in brood. Thus far, little is known on whether glochidia continue to be affected by external stressors after full encystment on host fish. Generally, a cyst consists of an epithelial canopy of varying thickness surrounding the larvae which are partially embedded in the gill filaments (Arey, 1932, Meyers et al., 1980, Howerth and Keller, 2006). To mimic natural conditions, host fish in our study remained in CEC treated water after infestations thus providing additional routes of exposure. While some evidence suggests that low levels of contaminants in the water do not influence fully encysted glochidial metamorphosis (Rach et al., 2006), more research is still recommended to conclude on the protective nature of the cyst and influence of continued host exposure (Cope et al., 2008).

Previous contaminant studies indicate glochidia exposure as a determinant of transformation success when maternal and host fish exposure are uncoupled from glochidia exposure. When unexposed glochidia from a variety of unionid species were allowed to encyst on host fish exposed to heavy metals above the lethal concentrations for glochidia, transformation rates were not impacted (Jacobson et al., 1997). This indicates that host fish exposure may not impact glochidia. In contrast, glochidia performance (i.e., viability and attachment) decreases after exposure to pollutants such as perfluoroakly acids and road salts when naïve, unexposed host fish are infested, demonstrating glochidial exposure maybe an important driver, uncoupled from host fish exposure, of transformation declines (Hazelton et al., 2012, Beggel and Geist, 2015, Gillis et al., 2021). If glochidia exposure is determined to be the primary detriment to transformation, more diligent monitoring in areas of vulnerable populations may be needed as CEC presence is often ephemeral. For example, in urbanized areas, a storm event could greatly increase the level of detectable CECs in runoff and landfill leachate (Masoner et al., 2014, Richardson and Ternes, 2014). In more rural environments, seasonal application of fertilizers may lead to unionid exposure after spring rains in addition to lower levels that persist in water and sediment (Carafa et al., 2007). Our medium CEC concentration treatments were based on the current maximum CEC concentrations in the Great Lakes Region, but high and low concentrations may not be uncommon given the fluctuations in CEC entry to waterways. Therefore, organisms may experience a range of concentrations varying over time.

In addition to the diminished transformation rate, a shift to a later date of juvenile drop off were detected for all CEC treatments though overlap with control groups was noted (Table 6, Dunn’s test). Exposure to contaminants has been shown to delay attainment of developmental endpoints in both invertebrates and vertebrates with increased time to hatch and metamorphosis as well as increased deformity rates and lower viability (Pisa et al., 2015, Hayes et al., 2010, and Jezierska et al., 2009). It is unclear if the same mechanisms may be at play in unionids though individuals metamorphosing at earlier or later dates have been correlated with lower survivorship (Hanlon and Neves 2006). Shifts in peak drop off day were small (~ 4 to 7 days based on estimates of response curves) but not necessarily ecologically negligible should there be fitness differences in juveniles that were not quantified in this study. The extended duration of encapsulation may indicate an underlying change in metamorphosis based on initial lower glochidia quality, disruption throughout the transformation process as documented for other taxa, or an evolutionary mechanism in which the parasitic stage is lengthened to increase later chance of survival. Therefore, either increased or decreased juvenile fitness may result from this change in metamorphosis duration. Environmental stress has been implicated in metamorphosis retardation and diminished fitness of other bivalve species with free floating larvae (Wacker and von Elert 2002, Phillips 2004). Within unionids, glochidia of a variety of species are known to remain encysted for longer durations and metamorphose at higher rates when water temperatures are low (Roberts and Barnhart 1999). Though higher transformation was implied to be a response to reduced host immune system, it has also been proposed glochidia in related freshwater bivalve family Margaritiferidae that glochidia which remain encysted longer are more likely to survive stressful conditions as juveniles excise at a larger size (Marwaha et al. 2017); this growth has never been observed in Unionidae and would take detailed microscopy to answer. Additional studies on the changes in juvenile quality in response to CEC exposure transformed juveniles should be considered. The information presented here encourages future investigations of stress impacts on juvenile transformation to include metrics to assess probable juvenile success after drop off.

Despite timing of juvenile drop off, the most successful treatments in numbers of transformed juveniles were the control and intermediate concentration agricultural mixture treatments with lowest numbers of metamorphosed individuals in low and high concentration urban mixtures. For these particular CEC mixtures, there may be synergistic or antagonistic effects present that could lead to detrimental effects to transformation at lower and higher concentrations for both agricultural and urban mixtures. Hormetic responses, wherein intermediate doses may be stimulating or therapeutic, are common among chemical stressors and could be at play (reviewed in Calabrese and Baldwin 2001, Agathokleous et al. 2021). Hormetic responses contrast with traditional thought on linear impacts of stressor concentration. Either hypothesis could lend itself to the dampened transformation of juveniles by CE treated host fish. The ethanol concentration in the solvent control (CE) and CEC treatments was nearly two hundred times less concentrated than standard protocols for shorter duration early life stage unionid studies (ASTM International 2013) and should have allowed for comparisons between CEC treatments and the effect of the solvent. However, the solvent control (CE) resulted in low transformation success. Given the complexity of presented mixtures (Table 1), we are unable to predict if antagonistic interactions masked the effects of ethanol alone. It is unclear if nonlinear responses to the solvent may occur at low doses previously believed to be below effective concentrations as is seen in U-shaped hormetic responses. Given the effects seen within our study, current protocols may need to be revised for chronic studies of longer duration or different life stage evaluations.

Success in agricultural treatments relative to urban treatments may indicate unionid acclimation to CECs at the collection site. The collection site for the unionids in this study at the Grand River, MI is dominated by agricultural land use (MDEQ, 2011). Thus, *L. cardium* from the collection site may have been acclimated to the same types of chemical stressors at similar concentrations to the intermediate agricultural treatment, allowing for higher transformation success in AM in comparison to UM where contaminants may be novel to the organisms. However, past models of habitat suitability have noted negative impacts of urban land use on multiple species of unionids (Daniel et al., 2018), and surveys of streams comparing areas of urbanization to areas of less developed have seen a decrease in unionid diversity in urbanized areas (Krebs et al., 2010). The decrease in diversity and unionid presence in urbanized regions may be indicative of heightened sensitivity to urban pollutants, though it is important to note that such changes in diversity may also be the result of other habitat alterations associated with developed land use or other stressors (Newton et al., 2008, Downing et al., 2010).

Even in agricultural treatments which had lower impacts on transformation than urban treatments, population level projections demonstrated an alarming reduction in growth rate (Fig. 3) leading to potential extirpation under continued CEC exposures. This illustrates that even small reductions in transformation as seen with these exposures may be of particular concern for rare species, and declining or stressed populations for which such reductions in recruitment could be devastating. The transformation from larval glochidia to juveniles was found to have the highest elasticity, indicating that even a slight percent change in transformation will have the largest proportional effect on population size, while adult fecundity had the highest sensitivity among life stages. The results of elasticity and sensitivity analysis indicates that management may intervene at two stages: the number of glochidia stocked (related to the elasticity of adult fecundity) or mitigating transformation success (related to the sensitivity of juvenile transformation). While hatcheries may provide additional future glochidia stock in the form of individual mussels, it may be difficult if waters are highly contaminated, as in AH and UH treatments, to overcome the low transformation success noted in this study. Furthermore, contaminant concentrations and thus exposures experienced by glochidia are often ephemeral. While medium concentrations were based on maximum concentrations measured in the Great Lakes region, we must note that elevated concentrations as seen in AH and UH are possible during weather events that alter flow, seasonal CEC use as in pesticides, among other mechanisms (e.g., Fairbairn et al., 2016). Therefore, conservation of unionids must prioritize areas of low(er) contamination for augmentation and recovery efforts to enable more successful natural reproduction and recruitment. However, we do caution that data used to parameterize the Lefkovich matrix resulted in a lambda value (annual population growth rate) just over 1, representing a stable population. While our lambda value was parameterized based on real data of both observational and manipulative studies (Table 4), we must recognize that some populations may have higher or lower growth rates. In populations with higher growth rates that are increasing population size, the introduction of CECs similar to the AM mix may not lead to the dramatic declines projected in our study but rather a modest decrease in growth. Given the imperilment of the unionid taxa, we note that decreases in population growth in healthy populations may still have far reaching effects through metapopulation dynamics and understanding population and metapopulation growth structures has been identified as a need for further research (Ferreira-Rodríguez et al., 2019).

While the precise influence of CECs on unionid juvenile transformation requires further exploration, a few variances are clear from this study: even a short period of exposure to ecologically relevant CECs may reduce the number of successfully transformed juveniles and lead to a shift in the duration of encapsulation. Limiting chemical exposures are therefore an important factor in reducing recruitment loss. Management efforts which minimize exposures of chemicals associated with wastewater treatment, sewage overflows and other urban developed area point sources, which had the highest impact in our study, could be especially valuable to unionid conservation. Additionally, point source and non-point source reduction efforts may have the highest conservation value if seasonally focused to avoid exposure during the periods of glochidia release when the larvae may be most vulnerable. Mitigation against chemical exposure must be considered, particularly during the highly sensitive larval and transformation life stages, leading to restricted recruitment and reduced populations or species loss.

## Supplementary information

Supplementary information
