# Correction: Alterations to unionid transformation during agricultural and urban contaminants of emerging concern exposures

**DOI:** 10.1007/s10646-023-02661-8

**Published:** 2023-05-10

**Authors:** Lacey D. Rzodkiewicz, Mandy L. Annis, Daelyn A. Woolnough

**Affiliations:** 1 Department of Biology and Institute for Great Lakes Research, Central Michigan University, 1455 Calumet Ct., Mt. Pleasant, MI 48859 USA; 2 US Fish & Wildlife Service, Michigan Ecological Services Field Office, 2651 Coolidge Road, Suite 101, East Lansing, MI 48823 USA; 3 Present Address: Department of Biological Sciences, University of Pittsburgh, 4249 Fifth Ave, Pittsburgh, PA 16509 USA

Correction to: Ecotoxicology (2023)

10.1007/s10646-023-02645-8

In the original publication of the article, the author noticed an error in the supplementary link. The wrong supplementary files were originally published which has now been replaced with the correct file with this correction.

The original article has also been corrected.

## Supplementary information

Supplemental Information

