# Supplementary material for: Alterations to unionid transformation during agricultural and urban contaminants of emerging concern exposures
Source: Ecotoxicology. 2023 Apr 20;32(4):451–68. doi: 10.1007/s10646-023-02645-8 (PMC10199875; doi:10.1007/s10646-023-02645-8)
Supplement: Supplementary file 1 — Supplementary information [file 10646_2023_2645_MOESM1_ESM.pdf]

Supplemental Information

Supplemental Information Table 1. Water quality data ($\bar{x} \pm SD$) throughout study for each treatment (CW: water control, CE: ethanol control, A: Agricultural, U:Urban, L:Low, M:Medium, H:High). *

Year	Treatment	pH	DO (%)	Temp (˚C)	Ammonia (ppm)	Alkalinity (ppm)
**2017**	**CW**	7.51 ± 0.28	93.63 ± 3.33	20.27 ± 1.47	0.09 ± 0.11	60.67 ± 23.80
	**CE**	7.90 ± 0.25	92.23 ± 8.93	19.59 ± 0.77	0.17 ± 0.06	65.00 ± 14.55
	**AL**	7.43 ± 0.33	91.57 ± 3.28	19.82 ± 1.20	0.09 ± 0.09	57.67 ± 15.42
	**AM**	7.39 ± 0.29	93.09 ± 11.34	19.13 ± 2.24	0.11 ± 0.11	56.00 ± 12.00
	**AH**	7.49 ± 0.32	91.56 ± 4.46	19.74 ± 1.36	0.12 ± 0.09	60.33 ± 28.58
	**UL**	7.83 ± 0.25	89.85 ± 8.81	19.66 ± 0.86	0.13 ± 0.03	71.38 ± 25.87
	**UM**	7.78 ± 0.19	90.29 ± 7.82	19.48 ± 0.96	0.14 ± 0.05	76.33 ± 16.43
	**UH**	7.80 ± 0.19	90.29 ± 7.82	19.48 ± 0.96	0.14 ± 0.05	76.33 ± 16.43
**2018**	**CW**	7.60 ± 0.32	90.87 ± 4.93	21.63 ± 0.53	0.12 ± 0.06	68.00 ± 11.94
	**CE**	7.71 ± 0.32	86.20 ± 7.47	20.37 ± 0.47	0.13 ± 0.05	69.67 ± 13.29
	**AM**	7.66 ± 0.27	86.47 ± 5.11	21.05 ± 0.64	0.12 ± 0.05	68.33 ± 13.19
	**UM**	7.71 ± 0.35	86.19 ± 6.52	20.56 ± 0.55	0.12 ± 0.04	67.00 ± 12.69

*Total chlorine and free chlorine were recorded as "0" across both years and all sampling events.

Supplemental Information Figure 1.

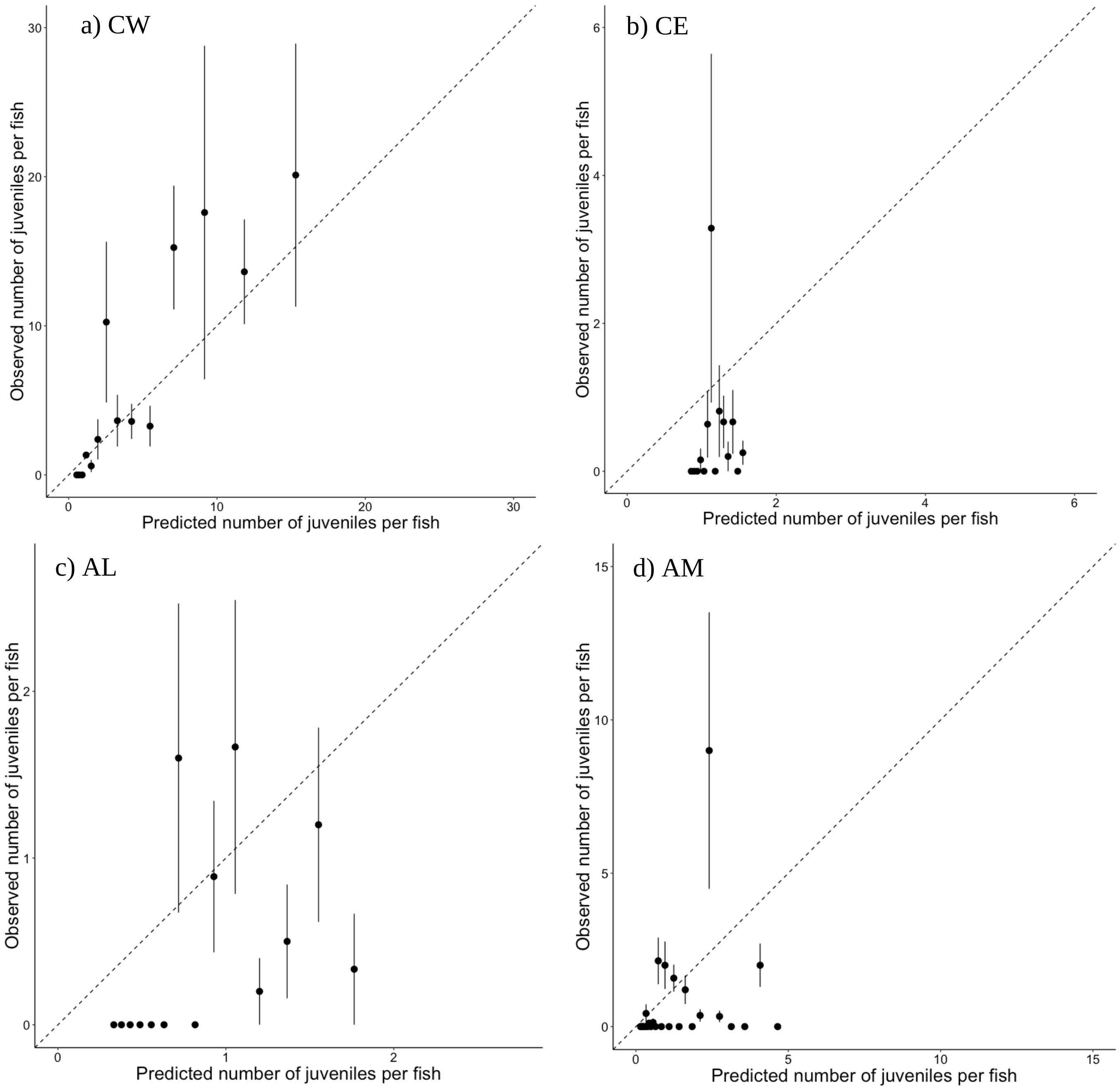

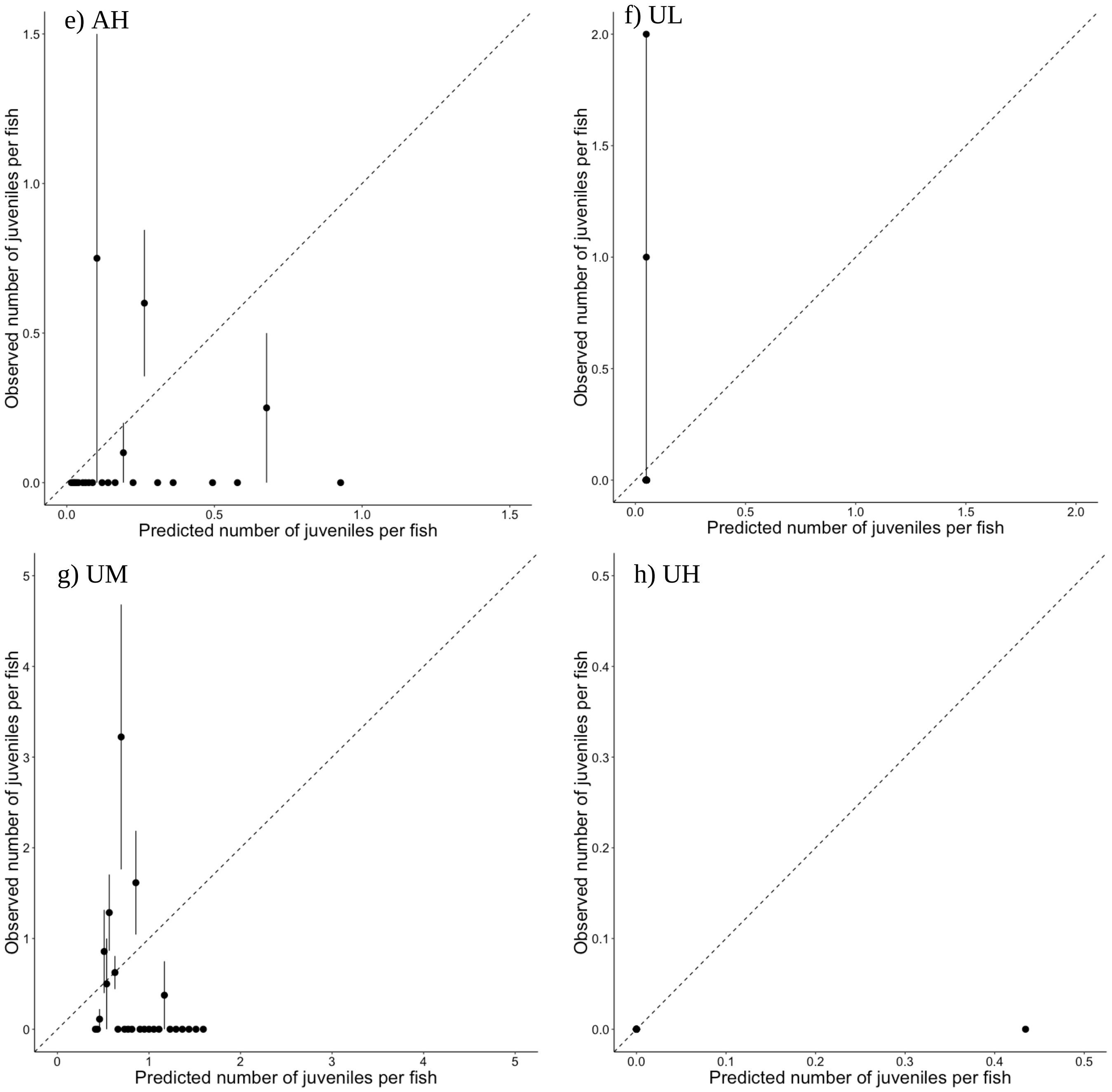

Supplemental Information Figure 1. Predictions of *Lampsilis cardium* juveniles transformed per *Micropterus salmoides* on a given day post-infestation for the test dataset (filter quantifications=1179) using population averaged random effects. Moderate fit was achieved across all treatments (R^2=0.287). Predictions are shown for each control, water (CW;a) and ethanol (CE; b), agricultural mixture in low (AL; c), medium (AM; d), and high (AH; e) concentrations, and urban mixture in low (UL; f), medium (UM; g), and high (UH; h) concentrations.
